# Small muscle mass aerobic exercise in health and disease: Unique insights into muscle vascular and metabolic control and performance

**DOI:** 10.1113/EP093475

**Published:** 2026-05-15

**Authors:** Shunsaku Koga, David C. Poole, Thomas J. Barstow, Dai Okushima, Richie P. Goulding

**Affiliations:** ^1^ Applied Physiology Laboratory Kobe Design University Kobe Japan; ^2^ College of Health and Human Sciences and Department of Anatomy and Physiology Kansas State University Manhattan Kansas USA; ^3^ Information and Management Systems Engineering Nagaoka University of Technology Nagaoka Niigata Japan; ^4^ Department of Human Movement Sciences, Faculty of Behavioral and Movement Sciences, Amsterdam Movement Sciences Vrije Universiteit Amsterdam Amsterdam The Netherlands

**Keywords:** critical power, exercise tolerance, knee extension exercise, near infrared spectroscopy, O_2_ delivery‐utilization matching, small muscle mass exercise, ${\dot{V}}_{O_{2}}$ kinetics

## Abstract

Studies of small muscle mass exercise (SMME) have revealed that the peripheral O_2_ transport–utilization cascade is a dynamically regulated system in which perfusive and diffusive components can be selectively amplified, redistributed and mechanically limited depending on contraction pattern, recruitment strategy and intramuscular pressure development. By relatively unbridling skeletal muscle from systemic circulatory restraint, SMME can expose both the maximal capacity and the intrinsic mechanical vulnerabilities of convective and microvascular O_2_ delivery (${\dot{Q}}_{O_{2}}$) in humans. The evidence reviewed herein demonstrates that ${\dot{V}}_{O_{2}}$ kinetics in young healthy individuals, performing cycling or SMME, are not limited by ${\dot{Q}}_{O_{2}}$ per se. With mass‐specific blood flows and diffusive conductance being markedly elevated during SMME, intramuscular metabolic regulation remains the dominant determinant of ${\dot{V}}_{O_{2}}$ kinetics, while alterations in ${\dot{Q}}_{O_{2}}$‐to‐${\dot{V}}_{O_{2}}$ matching primarily shape fatigue development, metabolite accumulation and force economy rather than the speed of the ${\dot{V}}_{O_{2}}$ kinetics. SMME alters not only the pattern of muscle recruitment but also the balance between ${\dot{Q}}_{O_{2}}$ and ${\dot{V}}_{O_{2}}$ in a muscle‐specific manner, uncoupling activation from deoxygenation in selected muscles. Above critical power, mechanical impedance to both conduit and microvascular blood flow can emerge, linking peripheral perfusion directly to metabolic instability and exercise intolerance. By isolating active muscle mass, SMME reveals whether a mitochondrial and microvascular metabolic reserve exists at peak exercise, and whether this reserve can be restored, or at least improved, through focused training. From a translational perspective, SMME provides a uniquely powerful framework to both diagnose and therapeutically target peripheral limitations in patients whose exercise intolerance is traditionally ascribed to central or ventilatory constraints.

## Abstract (highlights)

- **What is the topic of this review?**
  How reducing the active muscle mass affects O_2_ delivery–utilization (${\dot{Q}}_{O_{2}}/{\dot{V}}_{O_{2}}$) matching and what the mechanisms responsible are.
- **What advances does it highlight?**
  Knee extension exercise (KE) improves ${\dot{Q}}_{O_{2}}/{\dot{V}}_{O_{2}}$ matching as well as enhancing blood–myocyte diffusive O_2_ conductance, permitting greater mass‐specific metabolic rates. KE results in a more spatially homogeneous distribution of ${\dot{Q}}_{O_{2}}$ relative to ${\dot{V}}_{O_{2}}$ compared with cycling, and reflects altered muscle recruitment patterns and a redistribution of perfusive and diffusive O_2_ conductances that favours a higher and more uniform microvascular $P_{O_{2}}$ across both superficial and deep muscle regions.

## 1 INTRODUCTION

Whole body or large muscle mass exercise such as cycling or running is essential for defining the upper limit(s) for the integrated functioning of the O_2_ transport pathway (i.e., maximal O_2_ uptake, ${\dot{V}}_{O_{2}\max}$; Wagner, 2023) and also critical power (CP, the highest power output sustainable in a metabolic steady‐state), together with the curvature constant *W*′, representing the finite work capacity that can be performed above CP (Caen et al., 2024; Jones et al., 2010). However, the presence of a finite cardiac output and the necessity for tight control of systemic blood pressure dictate that, during cycling or running, the sympathetic nervous system constrains vascular dilation and thus prevents expression of the metabolic potential – the sleeping giant – of individual skeletal muscles and muscle groups (Andersen & Saltin, 1985; Joyner, 2004; Richardson et al., 1993). Thus, to unleash the sleeping giant and the full capability of individual muscles to sustain power and utilize O_2_ in health and disease, recourse must be made to small muscle mass exercise (SMME).

**FIGURE 1:**
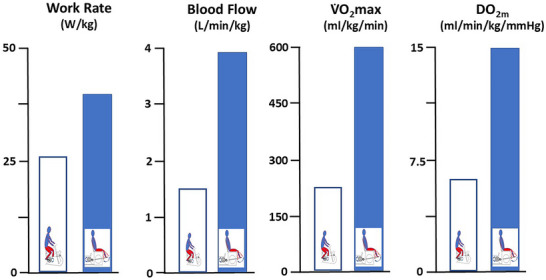
Comparison of maximal cycling (white bars) and knee‐extension (blue bars) exercise with respect to mass‐specific work rate, blood flow, maximal O_2_ uptake (${\dot{V}}_{O_{2}\max}$) and estimated muscle O_2_ diffusing capacity (${D_{O_{2}}}_{m}$). Notably both cohorts were well‐trained individuals with mean ${\dot{V}}_{O_{2}\max}$ values ∼60 mL/min/kg. Constructed using data from Richardson et al. (1993).

SMME refers to dynamic exercise performed with a limited absolute active muscle mass, typically involving a single limb or an isolated muscle group. Crucially, during SMME of locomotory or respiratory muscles, submaximal cardiac outputs do not necessarily invoke obligatory sympathetic restraint over individual muscle(s) and yet the fundamental characteristics of the power–time relationship and parameters CP and *W*′ still dictate performance (Jones et al., 2010; Poole et al., 2021). Even when a small muscle mass is engaged, exercise can elicit reflex cardiovascular adjustments mediated by muscle afferents, alterations in autonomic tone and redistribution of blood volume. Thus, local perfusion pressure and systemic haemodynamic regulation remain operative, although the ceiling imposed by maximal cardiac output is less likely to be reached compared with large‐muscle‐mass exercise. This relative unbridling of skeletal muscle(s) from central constraint allows far greater mass‐specific work output, muscle blood flow (${\dot{Q}}_{m}$), ${\dot{V}}_{O_{2}\max}$ and muscle diffusing capacity ($D_{O_{2}}$) as evidenced in Figure 1 when knee extension exercise (KE) is used as an exemplar of SMME versus conventional cycling (2.4 vs. ∼15 kg active muscle) (Richardson et al., 1993).

Nevertheless, the capacity for O_2_ flux per unit muscle is not limitless. In sedentary, older individuals, or peripherally deconditioned patient populations, without a substantial mitochondrial excess capacity at maximal exercise for conventional cycling, SMME can drive mitochondrial oxidative phosphorylation to its ceiling (Broxterman et al., 2024).

This review uses KE principally, but not exclusively, as an exemplar of SMME in which time‐resolved near infrared spectroscopy (TRS‐NIRS) has been used to specifically investigate spatial and depth heterogeneity of vascular and metabolic capacities among the quadriceps muscles during exercise (e.g., Koga et al., 2019; Okushima et al., 2020).

TRS‐NIRS employs pulsed light with sub‐nanosecond temporal resolution to determine absorption and scattering coefficients and mean photon path length, allowing calculation of absolute concentrations of deoxy[Hb+Mb], oxy[Hb+Mb] and total[Hb+Mb].

In particular, analysis of the temporal profiles of muscle [Hb+Mb] deoxygenation using TRS‐NIRS offers original insights into local ${\dot{Q}}_{O_{2}}$–${\dot{V}}_{O_{2}}$ matching, and because [Mb] does not change with exercise, changes in total[Hb+Mb] are considered reflective of changes in muscle $D_{O_{2}}$ (Barstow., 2019). Spatial and depth‐related heterogeneity in muscle deoxygenation and total [Hb+Mb] is also discussed as a mechanistic window into the regulation of local O_2_ delivery–utilization matching.

## 2 PHYSIOLOGICAL MECHANISMS

Single‐ or two‐leg KE has become the archetypal SMME paradigm for interrogating peripheral O_2_ transport and utilization, offering excellent compatibility with NIRS, EMG and/or diffuse correlation spectroscopy (DCS) alongside straightforward access to the femoral vessels, making it uniquely suited to isolate local vascular and metabolic responses when freed from central constraints. For these reasons, KE is used throughout this review as the primary exemplar of SMME, though single‐leg cycling, arm ergometry, handgrip and respiratory muscle exercise are also considered, with clinical and rehabilitative applications discussed in a later section. This focus reflects the availability of high‐resolution physiological data from this model, but it necessarily limits the extent to which the conclusions can be generalized to all forms of SMME, particularly those involving alternative small‐muscle‐mass configurations.

### 2.1 Kinetics of peripheral vascular and metabolic function (dynamic ${\dot{Q}}_{O_{2}}/{\dot{V}}_{O_{2}}$)

A central question in SMME physiology is how reduced active muscle mass influences ${\dot{Q}}_{O_{2}}$–${\dot{V}}_{O_{2}}$ matching and its underlying mechanisms. NIRS‐derived muscle oxygenation reflects the balance between O_2_ delivery (${\dot{Q}}_{O_{2}}$) and utilization (${\dot{V}}_{O_{2}}$): that is, the ${\dot{Q}}_{O_{2}}/{\dot{V}}_{O_{2}}$ ratio [or inversely ${\dot{V}}_{O_{2}}$/${\dot{Q}}_{O_{2}}$ (expressed as changes in deoxy[Hb+Mb]) indicating fractional extraction]. Changes in total[Hb+Mb] are interpreted as reflecting muscle diffusive O_2_ conductance ($D_{O_{2}}$) changes (Poole et al., 2021), although absolute quantification is not possible and depends on several assumptions. Despite limitations such as dependence of measured haem concentrations on adipose tissue thickness and skin blood flow, when appropriate correction factors are employed, NIRS permits integrated assessment of perfusive and diffusive O_2_ transport.

**FIGURE 2:**
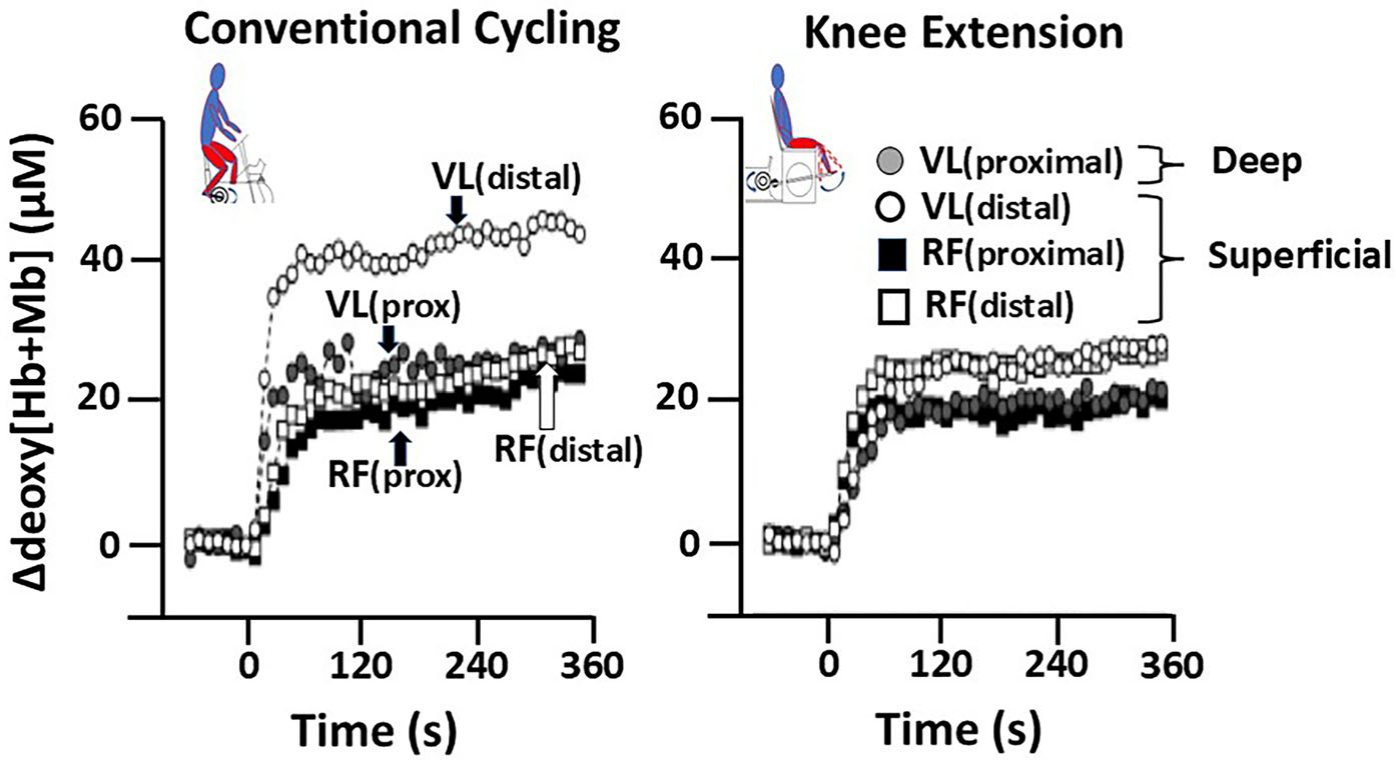
Group mean responses for muscle deoxygenation (deoxy[Hb+Mb]) from baseline to heavy exercise of CE and KE. Open and grey circles show responses for distal (superficial) and proximal (deep) vastus lateralis (VL). Open and filled squares show responses for superficial sites of distal and proximal rectus femoris (RF) (Koga et al., 2019).

**FIGURE 3:**
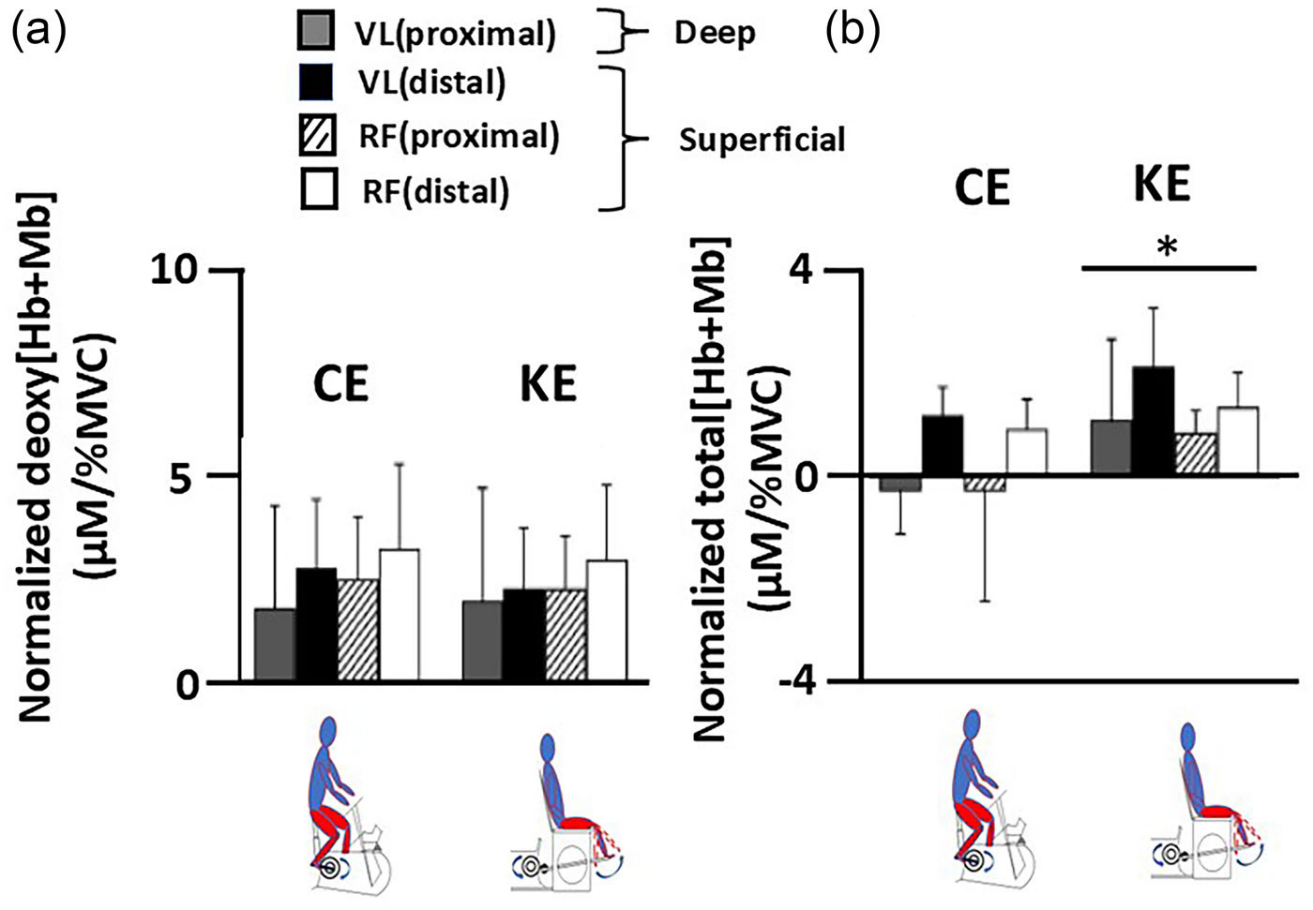
Group mean amplitudes of deoxygenation (deoxy) (a) and total[Hb+Mb] (b) normalized to the iEMG signal obtained during a maximal voluntary contraction [i.e., µM/% MVC] during heavy intensity exercise for cycle ergometry (CE) and knee extension ergometry (KE) for VL and RF. *Significant differences between KE and CE (Koga et al., 2019).

Koga et al. (2019) reported that, following heavy‐intensity exercise onset, superficial vastus lateralis (VL) deoxy[Hb+Mb] kinetics are slower and of lower amplitude during two‐leg KE (alternating kicking pattern) compared with cycling exercise (CE) (Figure 2), indicating higher ${\dot{Q}}_{O_{2}}/{\dot{V}}_{O_{2}}$ and better matching of microvascular O_2_ delivery to utilization. However, comparisons are complicated by differences in absolute power output (Koga et al., 2005). When normalized to integrated electromyography (iEMG, expressed as percentage of maximal voluntary contraction, %MVC), modality differences in deoxygenation are abolished (Figure 3), indicating that at equivalent levels of muscle activation, microvascular O_2_ extraction is independent of exercise mode across different muscle sites. This aligns with similar bulk leg O_2_ extraction in the two exercise modes (Richardson et al., 1993). Nevertheless, the greater increases in total[Hb+Mb] in VL and rectus femoris (RF) during KE compared with CE (Figure 3) suggest an enhanced blood–myocyte diffusive O_2_ conductance in KE.

Importantly, however, KE reveals distinct behaviour in muscles that are recruited minimally in CE but consistently more in KE. For example, during incremental exercise, the RF is not recruited to a large extent until higher fractions of peak work rate in CE (Okushima et al., 2020), whereas during KE the RF is recruited to a greater extent at lower relative work rates. Additionally, although Koga et al. (2019) observed that the majority of between‐muscle differences in deoxy[Hb+Mb] during heavy intensity exercise among KE and CE were abolished when normalized to iEMG, it remained unclear whether this was the case across the full spectrum of aerobically achievable work rates.

**FIGURE 4:**
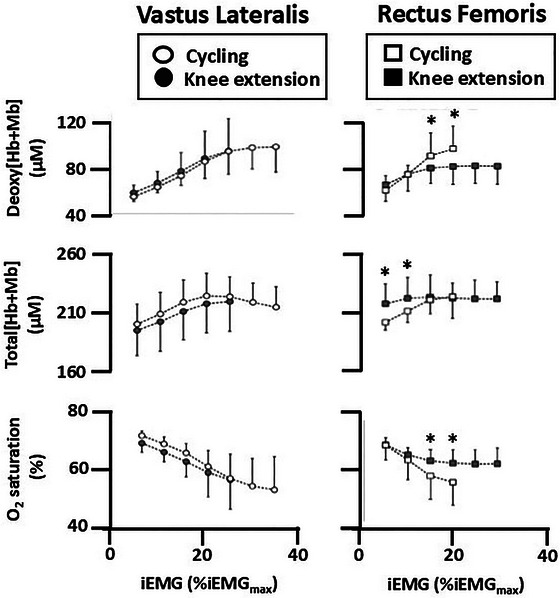
The relationship of near‐infrared spectroscopic measurements to the normalized integrated EMG (iEMG) level at each muscle site (vastus lateralis and rectus femoris). Top, deoxy[Hb+Mb]; middle, total[Hb+Mb]; and bottom, tissue O_2_ saturation. *Significant differences between exercise modalities at the same iEMG level (Okushima et al., 2020).

To address these issues, Okushima et al. (2020) examined NIRS‐derived muscle responses to incremental CE and KE as a function of iEMG as a percentage of MVC (iEMG_max_). The superficial VL deoxy[Hb+Mb] profile did not differ between CE and KE, implying a similar matching of ${\dot{Q}}_{O_{2}}$ to ${\dot{V}}_{O_{2}}$ in the VL for both exercise modalities. In contrast, the superficial RF exhibited lower deoxy[Hb+Mb] values during KE than during CE at 15% and 20% iEMG_max_. Conversely, total[Hb+Mb] in the RF during KE was greater than during CE at 5% and 10% iEMG_max_ (Figure 4), consistent with enhanced muscle effective $D_{O_{2}}$ and more favourable ${\dot{Q}}_{O_{2}}/{\dot{V}}_{O_{2}}$ at low work rates. Importantly, the RF was recruited to a greater extent during KE as compared to CE (i.e., mean iEMG at maximal exercise of 32.2 vs. 22.9%, respectively). However, despite this greater level of recruitment in the RF, deoxy[Hb+Mb] as a function of iEMG was essentially constant from 15% to 30% iEMGmax, and tissue O_2_ saturation was maintained higher over these levels of muscle activation. This suggests that KE unbridled tissue ${\dot{Q}}_{O_{2}}$ from metabolic rate (i.e., ${\dot{V}}_{O_{2}}$) in the RF, enabling greater ${\dot{Q}}_{O_{2}}/{\dot{V}}_{O_{2}}$ in this muscle despite its metabolic rate (inferred from iEMG measurements) exceeding that observed during CE. Thus, the differential deoxygenation profiles observed in the RF across exercise modalities may reflect muscle‐specific metabolic, vascular or architectural characteristics, and their associated haemodynamic responses.

In spite of the fact that ${\dot{Q}}_{O_{2}}$‐to‐${\dot{V}}_{O_{2}}$ matching appeared enhanced in the superficial RF, this was not necessarily the case during the transient phase of exercise. Specifically, at the superficial proximal RF, a faster primary component of deoxy[Hb+Mb] kinetics was observed during KE compared with CE (Koga et al., 2019). This finding may reflect a transient insufficiency of microvascular O_2_ delivery relative to metabolic O_2_ demand. Although this may seem difficult to reconcile with the findings of improved ${\dot{Q}}_{O_{2}}$‐to‐${\dot{V}}_{O_{2}}$ matching in the superficial RF by Okushima et al. (2020), it is important to note that these observations need not reflect the same underlying control mechanism. The faster deoxy[Hb+Mb] kinetics observed in the superficial proximal RF during KE (Koga et al., 2019) describe the rate at which microvascular O_2_ extraction adjusts at exercise onset, whereas the findings of Okushima et al. (2020) describe the magnitude of O_2_ extraction relative to muscle activation across the aerobic work‐rate spectrum. These two properties need not covary. KE typically involves longer relative contraction phases, which may induce repeated transient ischaemia. Lutjemeier et al. (2008) reported that the cyclical pattern of deoxygenation and oxygenation during the contraction–relaxation phases of KE suggests that oxygen extraction is not confined to the relaxation phase but continues during muscle contraction. Hence, given the higher and earlier recruitment of the RF during KE, it is plausible that elevated intramuscular tensions transiently accelerated local deoxygenation kinetics by mechanically constraining perfusion, even while the muscle operated with a higher perfusive and/or diffusive reserve overall. In contrast, cycling generally involves shorter contraction times and permits more continuous perfusion. Thus, a faster deoxygenation response does not preclude an uncoupling of O_2_ extraction from recruitment level, but rather indicates that distinct mechanical and vascular factors may differentially shape the kinetics and gain of ${\dot{Q}}_{O_{2}}$‐to‐${\dot{V}}_{O_{2}}$ matching in this muscle.

The dissociation of deoxygenation from activation implies that KE uniquely improves ${\dot{Q}}_{O_{2}}$‐to‐${\dot{V}}_{O_{2}}$ matching as well as enhancing blood–myocyte diffusive O_2_ conductance in selected muscles, permitting greater mass‐specific metabolic rates without invoking corresponding increases in muscle O_2_ extraction. Thus, SMME alters not only the pattern of muscle recruitment, but also the balance between perfusive and diffusive O_2_ conductance in a muscle‐specific manner.

### 2.2 Overall matching vs. heterogeneity of ${\dot{Q}}_{O_{2}}$ to ${\dot{V}}_{O_{2}}$ across and within muscles

Effective spatial ${\dot{Q}}_{O_{2}}$‐to‐${\dot{V}}_{O_{2}}$ matching during muscular contractions is essential as it determines microvascular $P_{O_{2}}$ and hence the upstream driving pressure for O_2_ diffusion, which in turn strongly influences intramuscular metabolic control. However, the ${\dot{Q}}_{O_{2}}/{\dot{V}}_{O_{2}}$ distribution among and within muscles is highly heterogeneous during CE (Koga et al., 2019), creating the potential for local O_2_ supply limitations otherwise concealed by single‐site NIRS measurements. Hence, this heterogeneity is physiologically meaningful, because this may, in turn, impede rates of mitochondrial ATP production, impair local metabolite homeostasis, and expedite exercise intolerance. Such observations can potentially inform our understanding of whether exercise tolerance is constrained uniformly across the active muscle mass or whether localized ‘weak links’ may contribute disproportionately to exercise limitation. Moreover, consistent with the notion that deep quadriceps muscle is more highly vascularized and oxidative than its superficial counterparts (Heinonen et al., 2015), we have repeatedly observed lower and slower deoxygenation (${\dot{V}}_{O_{2}}$/${\dot{Q}}_{O_{2}}$) profiles in deep compared with superficial muscle tissues during CE (Koga et al., 2015, 2019). However, whether this was also the case for KE remained unclear.

**FIGURE 5:**
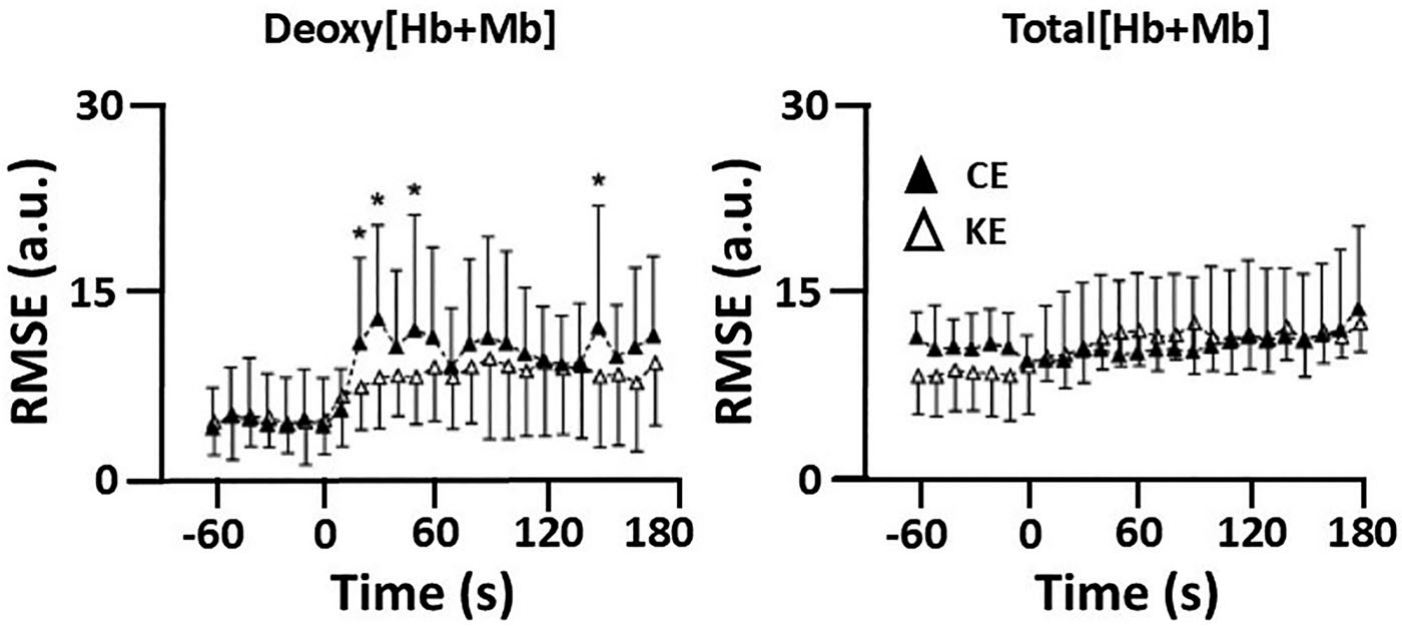
Group mean responses for root mean square error (RMSE) changes of muscle deoxy[Hb+Mb] and total[Hb+Mb] following the onset of heavy exercise. Open and filled triangles show the changes in KE and CE, respectively; a.u., arbitrary units. *Significant differences between KE and CE (*P *< 0.05) (Koga et al., 2019).

A lower degree of heterogeneity among selected quadriceps muscles was observed during KE compared with CE (Figure 5). The lower root mean square error across muscle sites, an index of spatial heterogeneity, of deoxy[Hb+Mb] during KE versus CE suggests a more ‘equal matching’ of ${\dot{Q}}_{O_{2}}$ to ${\dot{V}}_{O_{2}}$ across the quadriceps musculature. In addition, the deoxy[Hb+Mb] kinetics in the superficial VL were slower during KE compared with CE (Figure 2), suggesting that O_2_ delivery adapted more rapidly with respect to local muscle ${\dot{V}}_{O_{2}}$ at exercise onset, consistent with better matching of ${\dot{Q}}_{O_{2}}$ to ${\dot{V}}_{O_{2}}$ in the transient phase of exercise in KE. Hence, KE appears to result in a more spatially homogeneous ${\dot{Q}}_{O_{2}}$ to ${\dot{V}}_{O_{2}}$ compared to CE, along with higher ${\dot{Q}}_{O_{2}}/{\dot{V}}_{O_{2}}$ ratios in both the superficial and deep regions of the VL both at the onset of, and during, exercise. However, given that only two muscle sites were sampled by Koga et al. (2019), it remains possible that further heterogeneity in perfusive and diffusive O_2_ transport would have been present across the quadriceps or at different locations within the muscles interrogated.

Collectively, these findings indicate that KE results in a more spatially homogeneous distribution of ${\dot{Q}}_{O_{2}}$ relative to ${\dot{V}}_{O_{2}}$ across the quadriceps musculature compared with CE. This reflects not only altered recruitment patterns, but also a redistribution of perfusive and diffusive O_2_ conductances that favours a higher and more uniform microvascular $P_{O_{2}}$ across both superficial and deep muscle regions. Simultaneous determination of iEMG and NIRS‐derived muscle deoxygenation profiles has thus revealed that, for certain muscles (e.g. RF), there is a genuine uncoupling of recruitment from muscle O_2_ extraction, evincing elevated local ${\dot{Q}}_{O_{2}}$‐to‐${\dot{V}}_{O_{2}}$ ratios.

## 3 INFLUENCE OF SMME ON WHOLE‐BODY ${\dot{V}}_{O_{2}}$ KINETICS

Evidence from multiple SMME modalities reviewed below supports the contention that, in the upright position in healthy young individuals during both whole‐body exercise and SMME, ${\dot{V}}_{O_{2}}$ kinetics are not limited by O_2_ delivery. This fact does not preclude that, under certain conditions such as disease or ageing, ${\dot{V}}_{O_{2}}$ kinetics may become delivery‐limited.

### 3.1 Knee extension exercise

**FIGURE 6:**
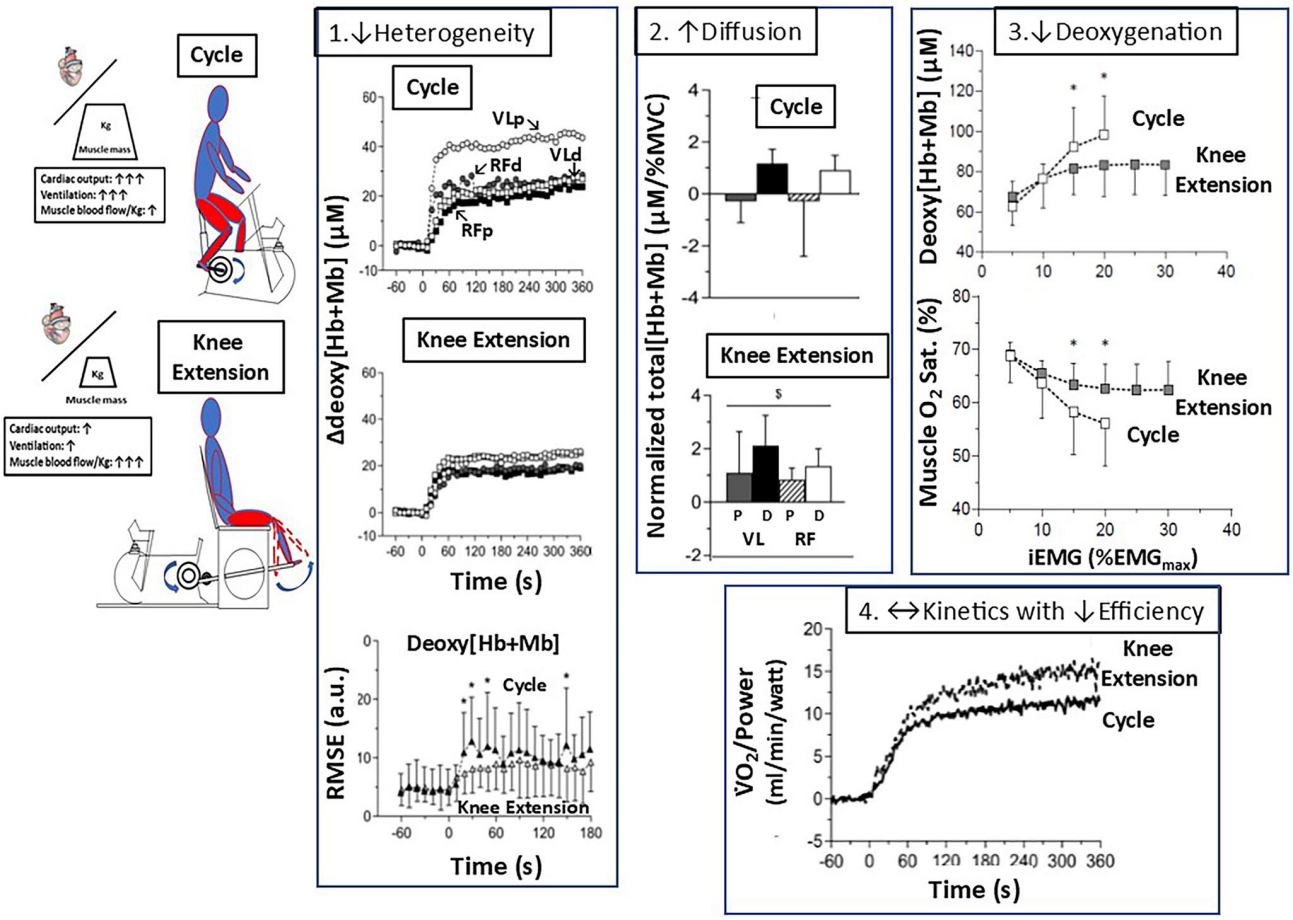
Spatial heterogeneity of vascular and metabolic capacities among the quadriceps muscles and pulmonary ${\dot{V}}_{O_{2}}$ response during knee extension exercise (an exemplar of SMME) compared with cycling. Analysis of the temporal profiles of muscle [Hb+Mb] deoxygenation offer insights into O_2_ delivery (${\dot{Q}}_{O_{2}}$)–${\dot{V}}_{O_{2}}$ matching and total[Hb+Mb]. See Figures 2, 3, 4, 5 for detail (Koga et al., 2019; Okushima et al., 2020) and Figure 1 in Koga et al., 2005. ${\dot{V}}_{O_{2}}$ kinetics for KE versus CE are not faster despite the presence of greater perfusive and diffusive O_2_ conductances across multiple sites within the quadriceps.

${\dot{V}}_{O_{2}}$ kinetics for KE versus CE are not faster despite the presence of greater perfusive and diffusive O_2_ conductances across multiple sites within the quadriceps (Koga et al., 2005, 2019) (Figure 6). This underscores that pulmonary and muscle ${\dot{V}}_{O_{2}}$ kinetics for both KE and CE in the upright position in young healthy individuals most likely reflect intramuscular limitations rather than limitations in O_2_ delivery.

### 3.2 One‐leg vs. two‐leg cycling exercise

If central O_2_ delivery limited the speed of the ${\dot{V}}_{O_{2}}$ kinetics during whole‐body exercise, then ${\dot{V}}_{O_{2}}$ kinetics should be speeded during one‐legged relative to two‐legged cycling, as the active muscle mass is halved during one‐legged cycling. Koga et al. (2001) demonstrated that the on‐transient ${\dot{V}}_{O_{2}}$ kinetics were indistinguishable across modalities during exercise. These findings indicate that, when muscle recruitment patterns are matched, ${\dot{V}}_{O_{2}}$ kinetics are independent of the active muscle mass, suggesting, once again, that central O_2_ delivery does not limit ${\dot{V}}_{O_{2}}$ kinetics.

### 3.3 Arm exercise

Arm exercise is widely used in wheelchair sports (e.g., basketball, rugby, tennis) and rehabilitation, providing a useful model to compare upper‐ and lower‐limb physiology. At exercise onset, pulmonary ${\dot{V}}_{O_{2}}$ kinetics during arm cranking exercise are consistently slower than during leg cycling (Koga et al., 1996). This likely reflects greater intermittent isometric contractions during arm cranking exercise, which mechanically compress the vasculature, attenuate the muscle pump, and restrict local perfusion, thereby limiting O_2_ delivery. In addition, arm muscles have a higher proportion of fast‐twitch fibres, which rely more on substrate‐level phosphorylation and may further slow ${\dot{V}}_{O_{2}}$ kinetics. While these mechanical and muscular factors likely contribute, it remains unclear whether whole‐muscle kinetics, local ${\dot{Q}}_{O_{2}}$‐to‐${\dot{V}}_{O_{2}}$ matching, and diffusive O_2_ conductance differ systematically from CE following exercise onset.

### 3.4 Integration across modalities

Although the elevated whole muscle and local ${\dot{Q}}_{O_{2}}/{\dot{V}}_{O_{2}}$ ratios afforded by SMME do not speed ${\dot{V}}_{O_{2}}$ kinetics, this does not imply that resultant $P_{O_{2}}$ elevations are unimportant for muscle contractile function. For example, hypoxic gas inspiration lowers intracellular $P_{O_{2}}$ (Broxterman et al., 2024) and increases metabolite accumulation (e.g., P_i_, H^+^, ADP) incurred at a given external work rate, whereas hyperoxic gas inspiration does the opposite (Haseler et al., 1998; Hogan et al., 1999). More recently, it was shown that the normal adjustment of ${\dot{Q}}_{O_{2}}$ to SMME was suboptimal for muscle contractile function, as when a metaboreflex‐initiated period of hyperperfusion was imposed during handgrip exercise, the ratio of muscle activation to force of contraction was improved (Drouin et al., 2023).

Collectively, these findings imply that even during SMME, where ${\dot{V}}_{O_{2}}$ kinetics are not O_2_ delivery‐limited, an elevated ${\dot{Q}}_{O_{2}}/{\dot{V}}_{O_{2}}$ ratio confers functional advantages by mitigating metabolite accumulation and improving force economy. This implies that interventions which enhance ${\dot{Q}}_{O_{2}}$‐to‐${\dot{V}}_{O_{2}}$ matching may improve exercise tolerance even in individuals whose ${\dot{V}}_{O_{2}}$ kinetics are not O_2_ delivery‐limited, because muscle contractile function and metabolite accumulation remain sensitive to microvascular $P_{O_{2}}$.

## 4 CRITICAL POWER AND *W*′

Although the most common application of the CP concept today is during large‐muscle mass, whole‐body exercise such as cycling or running, much of its foundational development stemmed from studies of SMME. As noted above, blood flow limitation for SMME is more likely associated with the contracting muscle(s) than with central (cardiac output) limitation. Specifically, intramuscular pressure during contractions compresses blood vessels, impeding muscle blood flow and increasing the likelihood that blood flow limitation will occur at high exercise intensities. Relevant to this review, how does blood flow limitation impact CP and the curvature constant (*W*′) for SMME? The concept of CP is based on exercise intensities which can typically be sustained for between 2 and 20 min, where a hyperbolic relationship between external power and the time to task failure (i.e., limit of tolerance, *T*_lim_) is observed. CP reflects the asymptote of the hyperbola, whereas *T*_lim_ above CP is defined by a constant amount of work (*W*′) and independent of actual power output. This constant *W*′ implies a similar or uniform mechanism for intolerance across this range of severe‐intensity work rates. Whilst CP and *W*′ are themselves determined phenomenologically, both parameters are determined by physiological processes (e.g., Goulding & Marwood, 2023; Poole et al., 2016). This section outlines how the SMME model has advanced our understanding of the mechanisms that underpin the CP concept.

### 4.1 Blood flow limitation of CP

At high contractile intensities during SMME, skeletal muscle blood flow is shaped by competing forces: vasodilation primarily of the inflowing arterioles and elevated arterial pressures coupled with the rhythmic action of the muscle pump facilitating venous return and increased arterial inflow, whereas elevated intramuscular pressures mechanically compress arterioles and capillaries, reducing convective O_2_ delivery. This dynamic makes SMME uniquely suited for understanding the role of muscle blood flow in shaping the power–duration relationship.

Exercise using a single leg KE ergometer demonstrated attenuated femoral artery blood flow (${\dot{Q}}_{\mathrm{fa}}$) responses at higher work rates during both supine (Hoelting et al., 2001) and upright (Lutjemeier et al., 2005) exercise. However, peak ${\dot{Q}}_{\mathrm{fa}}$, CP and *W*′ were not determined in those studies. To specifically evaluate the impact of contraction pattern‐induced blood flow limitations on CP and *W*′, Broxterman et al. (2014) employed duty cycles of 20% and 50% for handgrip exercise across several work rates. At the same absolute work rate, brachial artery blood flow (${\dot{Q}}_{\mathrm{ba}}$) by Doppler, deoxy[Hb+Mb] by NIRS and estimated muscle ${\dot{V}}_{O_{2}}$ were greater for the 20% duty cycle, as was CP, but *W*′ was not different. More recently, Hammer et al. (2020) determined that blood flow below critical force (CF, isometric equivalent to CP) increased as a function of force whereas above CF it plateaued, decreasing exercise tolerance proportionally with increasing force above CF. These data support the presence of a maximal limit to limb blood flow under these specific conditions and highlight the critical role of muscle perfusion in determining severe‐intensity exercise tolerance.

The finding of significant impedance to limb blood flow for exercise above, relative to below, CP in forearm exercise was replicated in near‐supine CE by Hammer et al. (2021). ${\dot{Q}}_{\mathrm{fa}}$ and vascular conductance (LVC) were measured by Doppler sonography in subjects cycling at power outputs below and above CP. During the first nine cardiac cycles of early recovery, ${\dot{Q}}_{\mathrm{fa}}$ and LVC increased significantly following work rates above, but not below, CP, implying that contraction‐induced impedance was present during the active >CP work rates.

Collectively, these studies, conducted primarily using SMME paradigms, demonstrate that (1) CP is highly dependent on intramuscular blood flow, and that (2) CP itself reflects a threshold above which muscle blood flow may become impeded for some exercise paradigms. This blood flow limitation likely acts in concert with the influence of intramuscular oxygen utilization on CP described elsewhere (Goulding & Marwood, 2023; Goulding et al., 2021), implying that the power–duration curve is influenced by the entire oxygen transport–utilization pathway. As a consequence, impaired blood flow above CP might contribute to the metabolic instability observed in the supra‐CP domain.

However, impedance or attenuation of limb blood flow above critical speed (CS) or CP is not universally observed in humans (e.g., KE exercise, Richardson et al., 1993) or animals. In particular, Copp et al. (2010) utilized labelled microspheres in rats running on a treadmill and determined that fast twitch (≥69% Type IIb/d/x) muscle blood flows >CS were far higher than predicted based upon those at CS. Whereas the discrepancy between these data and those cited earlier may potentially be explained by the relatively high percentage of glycolytic fibres/muscles in the rat compared to human skeletal muscle, that some of the highest muscle blood flows measured were in humans performing KE exercise (Richardson et al., 1993) implicates a crucial role for duty cycle, contraction frequency and likely distribution and magnitude of intramuscular pressures.

### 4.2 Distinction between conduit and microvascular flow

**FIGURE 7:**
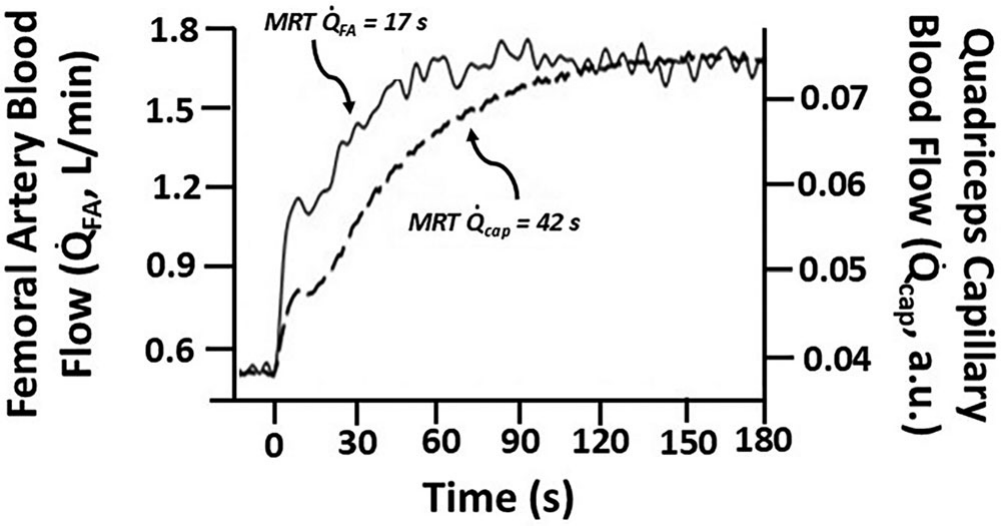
The kinetics of adjustment of capillary blood flow (${\dot{Q}}_{\mathrm{cap}}$) during moderate intensity KE exercise (Harper et al., 2006). During exercise the mean response time for femoral artery blood flow (${\dot{Q}}_{\mathrm{fa}}$) was significantly faster than that of ${\dot{Q}}_{\mathrm{cap}}$.

**FIGURE 8:**
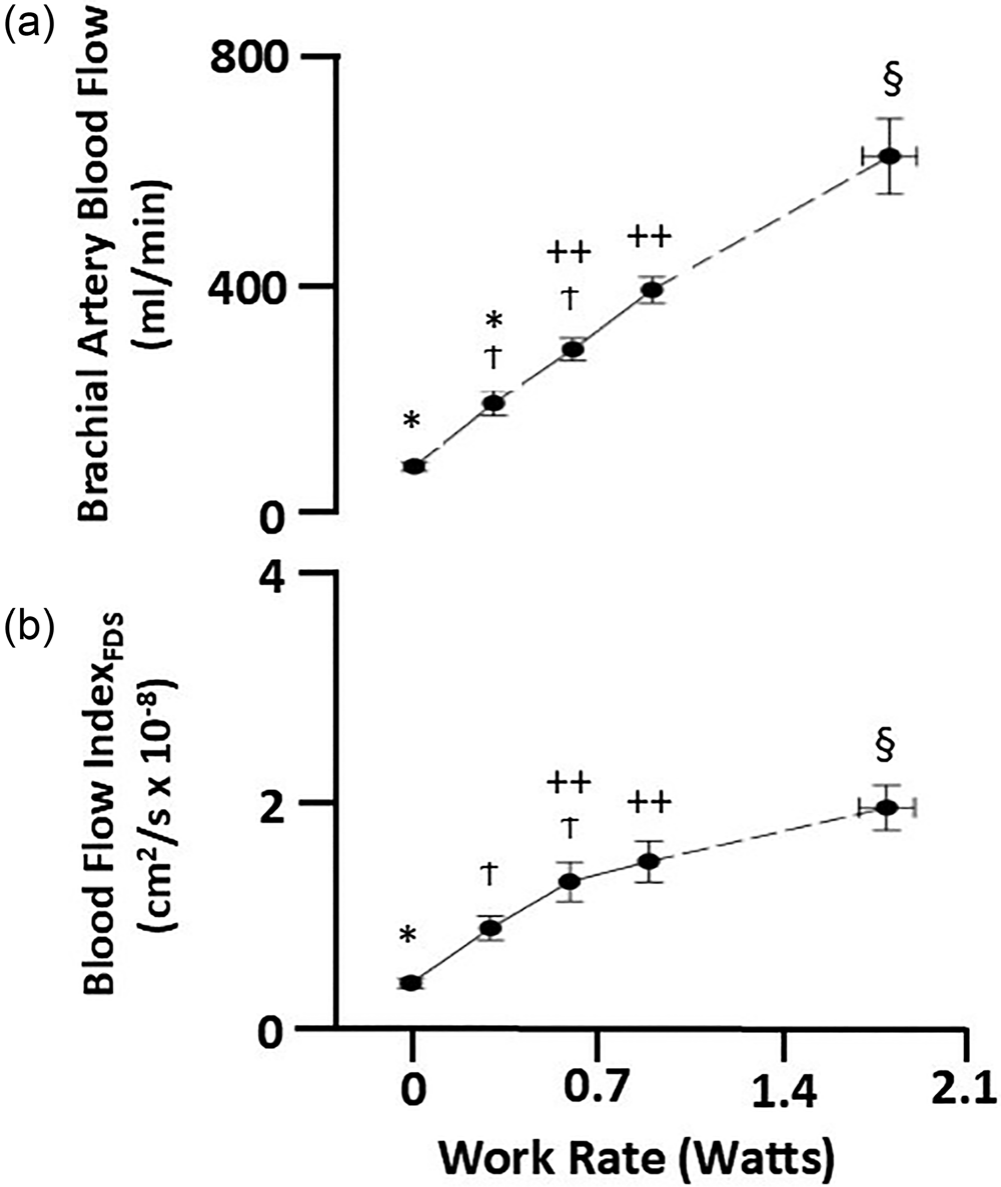
Bulk conduit artery and microvascular haemodynamic responses during the exercise protocol (Hammer et al., 2018). The mean responses for brachial artery blood flow (a) and blood flow index (b) of the flexor digitorum superficialis from rest to peak power during the incremental handgrip exercise test performed until task failure.

The discussion above regarding impedance to blood flow during SMME in humans focused on responses of limb blood flow at high exercise intensities. However, whether this impedance might also exist in the microcirculation, where blood–myocyte gas exchange ultimately occurs, was unknown. Previously, the kinetics of adjustment of capillary blood flow (${\dot{Q}}_{\mathrm{cap}}$) during moderate intensity KE exercise (Harper et al., 2006) (Figure 7) and in recovery (Harper et al., 2008) was estimated using pulmonary ${\dot{V}}_{O_{2}}$ and deoxy[Hb+Mb] as a surrogate for fractional O_2_ extraction. During exercise the mean response time for ${\dot{Q}}_{\mathrm{fa}}$ was significantly faster than that of ${\dot{Q}}_{\mathrm{cap}}$, with the kinetics of ${\dot{V}}_{O_{2}}$ residing somewhere between the two flows, suggesting that blood flow kinetics may be controlled differently at the microcirculatory versus arterial level. In contrast, during exercise recovery there were no differences among the kinetics of ${\dot{V}}_{O_{2}}$, ${\dot{Q}}_{\mathrm{cap}}$ and ${\dot{Q}}_{\mathrm{fa}}$. However, as the ${\dot{Q}}_{\mathrm{cap}}$ responses were not measured but calculated, and conflated local (deoxy[Hb+Mb]) with systemic (${\dot{V}}_{O_{2}}$) measures, it was necessary to more directly probe these responses. Thus, DCS was employed to estimate muscle microvascular blood flow non‐invasively and compare to ${\dot{Q}}_{\mathrm{ba}}$ during incremental handgrip exercise (Hammer et al., 2018) (Figure 8). Both the ${\dot{Q}}_{\mathrm{ba}}$ and muscle activation increased with exercise intensity until the last work stage, but the rise in blood flow index of the flexor digitorum superficialis and muscle oxygenation was attenuated by ∼50% peak work rate, demonstrating plateau behaviour. Together, these findings imply that limitations to perfusion during SMME may also arise at the microvascular level, where mechanical vessel compression may cap capillary blood flow and diffusional O_2_ conductance, even while bulk limb convective O_2_ transport and muscle activation continue to rise. Hence, this restricted microvascular response likely contributes to the loss of metabolic stability that is observed above CP. This implies that both the conduit and microvascular components of the O_2_ transport pathway contribute to shaping the power–duration relationship. Crucially, however, some of the very highest mass‐specific blood flows have been measured during SMME (Richardson et al., 1993), without clear evidence of submaximal plateaus. Whether this apparent discrepancy reflects methodological differences, exercise mode or the effect of training status is at present unclear. However, current techniques for assessing muscle ${\dot{Q}}_{\mathrm{cap}}$ during exercise in humans such as DCS are primarily restricted to superficial regions of muscle. Hence, the likelihood that deeper muscle tissues do not behave in a similar fashion during exercise above versus below CP should be resolved.

## 5 SMME AS A CLINICAL PARADIGM

SMME has proven invaluable as an investigative model and as a therapeutic modality for several chronic diseases. This section briefly reviews this approach and highlights the exciting role that SMME can play in stimulating peripheral adaptations in O_2_ transport and utilization therapeutically.

Exercise intolerance is a defining feature of both heart failure (HF) and chronic obstructive pulmonary disease (COPD) and was traditionally ascribed, almost exclusively, to ‘central’ causes: a malfunctioning central cardiac pump on the one hand, and pulmonary limitation on the other. However, mounting evidence in both diseases revealed structural and functional deficits in peripheral oxidative metabolism including reduced mitochondrial volume density and/or respiratory capacity (e.g., Gifford et al., 2018), muscle capillary rarefaction and compromised capillary haemodynamics (Jobin et al., 1998), and muscle atrophy (Mancini et al., 1992). Unfortunately, because large‐muscle‐mass exercise is centrally constrained in HF and COPD, the contribution of these deficits to exercise intolerance largely escaped resolution. When SMME is employed, however, the greater mass‐specific rates of blood flow and O_2_ consumption achieved reveals whether or not a ‘healthy’ muscle mitochondrial metabolic reserve exists at peak exercise.

Utilizing this logic, Esposito et al. (2010) investigated the bases for the lower ${\dot{V}}_{O_{2}\max}$ during CE and KE in HF compared to controls. Crucially, the lower KE ${\dot{V}}_{O_{2}\max}$ was driven by reduced muscle ${\dot{Q}}_{O_{2}}\left({\dot{Q}}_{O_{2}}m\right)$ and muscle $D_{O_{2}}$ associated with muscle capillary rarefaction and decreased mitochondrial volume density (Esposito et al., 2010). In a similar vein, Broxterman et al. (2020) compared subjects with COPD and healthy controls during single‐leg KE. Despite the SMME modality circumventing ventilatory constraints to exercise capacity, muscle ${\dot{V}}_{O_{2}\max}$ during SMME was reduced by ${\dot{Q}}_{O_{2}m}$ and muscle $D_{O_{2}}$ deficits (as for HF; Esposito et al., 2010). It was therefore concluded that O_2_ transport dysfunction at the level of the skeletal muscle greatly impairs exercise capacity in patients with both HF and COPD. The use of SMME to bypass centrally imposed O_2_ transport constraints was instrumental for unveiling the peripheral contributions to this impaired exercise capacity.

Given the limited range of power outputs that COPD and HF patients can sustain, large muscle mass exercise training may not provide an adequate muscle stimulus for skeletal muscle angiogenic and metabolic adaptations (Gouzi et al., 2013). Indeed, given its ability to achieve far higher mass‐specific muscle metabolic rates than whole‐body exercise, SMME training has been shown to potentiate muscle adaptations in both HF and COPD. Specifically, Esposito et al. (2011) determined that 8 weeks of double‐legged KE training in congestive heart failure patients, whilst not improving central cardiac function, increased maximal ${\dot{Q}}_{O_{2}m}$ 54% and muscle $D_{O_{2}}$ 39% during KE, driving a 53% improvement in single‐leg ${\dot{V}}_{O_{2}\max}$. Remarkably, post‐training ${\dot{V}}_{O_{2}\max}$ measured during CE in these HF patients had risen to match that in healthy, sedentary controls. Similarly, the post‐intervention values for mitochondrial volume density and various markers of capillarization in HF were restored to, or slightly above, controls. A similar approach in COPD patients elevated muscle $D_{O_{2}}$ 38%, again matching that of untrained controls (Broxterman et al., 2021). Surprisingly, however, training the COPD group, unlike for healthy controls, did not enhance their ${\dot{Q}}_{O_{2}m}$. Nonetheless, the improvement in muscle $D_{O_{2}}$ alone in these COPD patients restored ${\dot{V}}_{O_{2}\max}$ to 80% of the untrained control values. Collectively, these studies demonstrate that SMME can both identify and reverse peripheral limitations to ${\dot{Q}}_{O_{2}m}$ and muscle $D_{O_{2}}$ in disorders classically viewed as centrally constrained. In populations where central cardiac or ventilatory limitations restrict exercise tolerance, or where large‐muscle‐mass exercise provokes unwanted exertional symptoms, as in long‐COVID or myalgic encephalomyelitis/chronic fatigue syndrome patients (Appelman et al., 2024), SMME represents a potent therapeutic strategy to oppose peripheral deconditioning, restore exercise capacity and increase the quality of life and prognosis. These findings mirror those that have been observed for resistance training, which has been widely recommended as an effective therapeutic strategy to improve functional capacity, muscle mass, and quality of life in populations with heart failure, COPD, ageing‐related sarcopenia and metabolic disease (e.g., reviews by Glover & Phillips, 2010; Warneke et al., 2025). SMME may be viewed as an additional strategy to enhance exercise tolerance in such populations, either alone or in combination with resistance training. Carefully controlled clinical trials directly comparing SMME with conventional training paradigms are now indicated.

## 6 CONCLUSIONS

Studies of SMME have revealed that the peripheral O_2_ transport–utilization cascade is a dynamically regulated system in which perfusive and diffusive components can be selectively amplified, redistributed and mechanically limited depending on contraction pattern, recruitment strategy and intramuscular pressure development. Through a relative unbridling of skeletal muscle from systemic circulatory restraint, SMME exposes both the maximal capacity and the intrinsic mechanical vulnerabilities of convective and microvascular O_2_ delivery in humans. Herein we have evidenced that SMME is a mechanistically incisive experimental and clinical tool for dissecting, and improving, the regulation of human O_2_ transport, mitochondrial energetics, fatigue and exercise intolerance across health and disease.

## AUTHOR CONTRIBUTIONS

Shunsaku Koga conceived the idea and wrote the first draft of the manuscript. All authors revised the manuscript and approved the final version submitted for publication. All authors have read and approved the final version of this manuscript and agree to be accountable for all aspects of the work in ensuring that questions related to the accuracy or integrity of any part of the work are appropriately investigated and resolved. All persons designated as authors qualify for authorship, and all those who qualify for authorship are listed.

## CONFLICT OF INTEREST

No conflicts of interest, financial or otherwise, are declared by the authors.
